# Recent Advancements of Monotherapy, Combination, and Sequential Treatment of EGFR/ALK-TKIs and ICIs in Non–Small Cell Lung Cancer

**DOI:** 10.3389/fphar.2022.905947

**Published:** 2022-06-06

**Authors:** Dehua Liao, Lun Yu, Dangang Shangguan, Yongchang Zhang, Bowen Xiao, Ni Liu, Nong Yang

**Affiliations:** ^1^ Department of Pharmacy, The Affiliated Cancer Hospital of Xiangya School of Medicine, Central South University, Changsha, China; ^2^ Department of PET-CT Center, Chenzhou NO. 1 People’s Hospital, Chenzhou, China; ^3^ Lung Cancer and Gastrointestinal Unit, Department of Medical Oncology, The Affiliated Cancer Hospital of Xiangya School of Medicine, Central South University, Changsha, China

**Keywords:** combination treatment, sequential treatment, NSCLC, TKIs, ICIs

## Abstract

Lung cancer is the leading cause of cancer-related deaths with high morbidity and mortality. Non–small cell lung cancer (NSCLC) is the most common type of lung cancer, accounting for 85% of all cases. Fortunately, the development of molecular oncology provides a promising and effective therapeutic strategy for lung cancers, including specific gene mutations/translocations and immune checkpoints, with epidermal growth factor receptor (EGFR) common mutations first and anaplastic lymphoma kinase (ALK) translocations later as the targeted therapy and immune checkpoint inhibitors (ICIs) as immunotherapy. This review summarized the recent therapy advancements of TKIs and ICIs in NSCLC and focused on the clinical effect of combination or sequential treatment so as to provide the effective advice for the treatment of NSCLC.

## Introduction

Lung cancer ranks as a leading cause of cancer-related deaths worldwide. According to the latest International Agency for Research on Cancer report, there were an estimated 2.2 million new cases of lung cancer worldwide and approximately 1.8 million deaths in 2020 (Sung et al., 2021). In addition, clinical screenings for lung cancer at an early stage were not widely conducted in some areas, and most cases of lung cancer were diagnosed at later stages (Maemondo et al., 2010). As it is known to us, squamous cell carcinoma, adenocarcinoma, and large cell carcinoma are the common types of lung cancer, and non–small cell lung cancer (NSCLC) accounts for approximately 85% of all cases (Jamal-Hanjani and Spicer, 2012). The median survival time for patients with untreated metastatic NSCLC is only 4 to 5 months, with the 1-year survival rate of only 10%. In the last 20 years, traditional cytotoxic chemotherapy achieved a response rate of 20%–35% and a median survival time of 10–12 months for NSCLC (Rapp et al., 1988; Schiller et al., 2002; Ohe et al., 2007). Furthermore, patients treated with chemotherapy generally suffered from the risk of reduction of life quality. In recent decades, with the rapid development in tumor molecular biology, the mutations of the epidermal growth factor receptor (EGFR) and the abnormal fusion of the anaplastic lymphoma kinase (ALK) have been reported, which have been successfully applied in clinical settings as targeted therapies. During the past few decades, numerous small-molecule tyrosine kinase inhibitors (TKIs) were widely approved as targeted therapy in NSCLC, including EGFR-TKIs and ALK-TKIs. For EGFR-TKIs, there are three generations, such as first-generation EGFR-TKIs (gefitinib, erlotinib, and icotinib), second-generation EGFR-TKIs (afatinib and dacomitinib), and third-generation EGFR-TKIs (osimertinib, almonertinib, and furmonertinib). There are also three generations of ALK-TKIs, including first-generation ALK-TKIs (crizotinib), second-generation ALK-TKIs (alectinib and ceritinib), and third-generation ALK-TKIs (lorlatinib). In patients with EGFR or ALK-activating mutations, those who underwent EGFR-TKI and ALK-TKI therapies showed better survival than those with traditional platinum doublet chemotherapy. Most of the TKIs provide a longer progression-free survival (PFS), and the benefit of overall survival (OS) is also observed in some TKIs. Importantly, TKIs provide a high quality of life for patients with controllable side effects (Mok et al., 2009; Rosell et al., 2012; Yang et al., 2015; Mok et al., 2017; Shi et al., 2017; Wu et al., 2017). Although the emergence of TKIs is a milestone for the treatment of advanced NSCLC, inevitable resistance will be observed after a few months of targeted therapy (EGFR-TKIs and ALK-TKIs) (Ohashi et al., 2013). The third-generation EGFR-TKIs, such as osimertinib, not only could be used for EGFR sensitive mutations (deletion of exon 19 and L858R point mutations in exon 21) but also for the treatment of patients with p. T790M mutation, which are failed by the first- or second-generation TKIs. However, newly acquired resistance will develop after osimertinib is given to patients with p. T790M mutation so as to other third-generation TKIs. Hence, there is still an urgent need to evaluate novel strategies for the treatment of these patients.

Large numbers of studies have investigated the involvement of the tumor micro-environment (TME) and immune system in the development of tumors, which provided integral comprehension and recommendations for the preparation of therapeutic regimens (Pardoll, 2012). As is known, checkpoint molecules (inhibitory receptors on T cells) control T-cell activation to avoid inflammation and pathology over-expression (Baumeister et al., 2016). Subsequently, immune checkpoint inhibitors (ICIs) could block inhibitory receptors and provide a new way in anticancer treatment due to the mechanism of activation. In particular, programmed cell death protein 1 (PD-1) and PD-1 ligand 1 (PD-L1) signals would send negative signals to the immune system. The high expression of PD-1 implies that T cells are dying from unusual antibodies released from tumors and become dysfunctional (Pauken and Wherry, 2015). Numerous studies have reported that the immune function of dying PD-1 expression-positive T cells could be rescued by blocking the connection between PD-1 and its ligands in animal models with tumors and viral infection (Iwai et al., 2002; Hirano et al., 2005; Barber et al., 2006). It has been revealed that PD-1 or PD-L1 caused the death of T cells and the inhibitors of PD-1/PD-L1 showed higher efficacy and safety in various tumors, including renal cell cancer (RCC), lung cancer, and melanoma. As shown in previous random phase III clinical studies (KEYNOTE-024, KEYNOTE-042, KEYNOTE-189, KEYNOTE-407, IMpower 110, etc.), ICIs exhibit better efficacy and lower toxicity than traditional chemotherapy in advanced NSCLC (Horn et al., 2017). Existing evidence provides novel therapeutic strategy for advanced NSCLC patients, especially for those without the mutation of oncogenic drivers, although they have ever received chemotherapy or not (Reck et al., 2016).

ICIs are emerging as novel treatment modalities for a variety of malignancies. Some ICIs have been approved for the first- and second-line treatment of advanced NSCLC. In comparison with traditional chemotherapy, no matter as monotherapy or combined with standard chemotherapy, ICIs all could improve survival, but the additional toxicity associated with immunotherapy affects the quality of life for patients to a greater or less extent (Schiller et al., 2002; Reck et al., 2016; Gandhi et al., 2018). Furthermore, dual immunotherapy (CTLA-4 inhibitors combined with PD1/PD-L1 inhibitors) has also been widely explored in numerous malignant tumors. A previous study has shown that nivolumab combined with ipilimumab showed a better response rate than nivolumab monotherapy in NSCLC, while the number of patients with grade 3 or 4 treatment-related adverse events was less than the conventional chemotherapy arm (Hellmann et al., 2019). In addition, the EGFR-sensitive mutation is always accompanied by the induction of PD-L1 overexpression, which contributed to the development of immune escape (Akbay et al., 2013). Gefitinib combined with durvalumab showed 79% ORR and 95% DCR in advanced NSCLC, and alectinib combined with atezolizumab showed 86% ORR and 21.7 months PFS in advanced NSCLC (Gibbons et al., 2016; Kim et al., 2018). These inspiring phase I clinical trial results allowed ICIs to be investigated as a follow-up targeted therapy treatment with high efficacy and low toxicity after the emergence of acquired resistance or as a concurrent treatment with targeted therapies. Meanwhile, in actual clinical practice, the advanced NSCLC patients, especially those with a higher PS score, are often treated with immunotherapy or immunochemotherapy to fill the gap while waiting for the genetic test results.

Herein, we summarized the clinical effects of corresponding therapies of EGFR/ALK-TKIs and ICIs, including monotherapy, combination, and sequential therapy, and hopefully, the information provided from this study may be beneficial for the treatment of NSCLC patients now and in the foreseeable future.

## Targeted Therapy in NSCLC

Almost one-third of the patients missed the best time for surgery as the characteristics of lung cancer in the early-stage are not obvious, which resulted in the difficulty of early diagnosis. Since the 1970s, 20%–30% of patients with advanced NSCLC have achieved a reproducible objective response to platinum-based combination chemotherapy (Fossella et al., 2003; Azzoli et al., 2009). In addition to chemotherapy, anti-angiogenic drugs such as bevacizumab are used in combination with carboplatin or paclitaxel for the treatment of advanced NSCLC, which achieved a longer PFS and OS. However, due to the inevitable progression of NSCLC, patients have to adjust with the treatment plan (Lynch et al., 2004; Paez et al., 2004). With the rapid development in molecular biology during the past few decades, targeted therapy has become a promising option for the treatment of NSCLC, and TKIs are recommended as the first-line treatment scheme in patients with positive mutations. Although not all the patients could benefit from TKIs, the emergence of TKIs provided more good choices for the treatment of advanced NSCLC. In clinical practice, TKIs are not only used as monotherapy but can also be combined with multiple other regimens. In recent decades, accumulating studies have provided the complete comprehension for the application of EGFR-TKIs and ALK-TKIs, and targeted therapy is a promising option for NSCLC patients.

### Treatment With EGFR-TKIs

Recent studies have shown that the majority of spontaneous mutations in EGFR are carcinogenic because the naive EGFR pathway can maintain apoptosis of normal cells (Paez et al., 2004; Costa et al., 2007). The abnormal activation of the EGFR signaling pathway promotes cell proliferation, survival, and anti-apoptosis, which means cells are highly dependent on an active EGFR pathway for survival. In addition, the confirmation of EGFR gene mutation provides possible treatment sites and the possibility of individualized treatment for patients, which is likely to elucidate the therapeutic prospects of controlling tumor development by targeting specific molecules. In particular, two commonly known mutations in EGFR genes, deletion of exon 19 (45–50%) and L858R point mutations in exon 21 (45%–50%), are more likely to occur in East Asians and non-smoking NSCLC patients (Shigematsu et al., 2005; Sequist et al., 2007; Ladanyi and Pao, 2008). The first-generation EGFR-TKIs, such as erlotinib, gefitinib, and icotinib, are reversible EGFR inhibitors. Gefitinib and erlotinib were initially approved for the treatments of advanced NSCLC patients with disease progression after traditional chemotherapy and demonstrated good efficacy in early clinical trials. In the clinical studies of IPASS (Mok et al., 2009), WJTOG3405 (Mitsudomi et al., 2010), and NEJ002 (Inoue et al., 2013), gefitinib showed longer PFS and higher ORR than those with traditional chemotherapy in EGFR-positive mutation-advanced NSCLC patients. Interestingly, the study CTONG1304 mentioned that gefitinib even showed effectiveness in patients with a PD therapeutic effect in pre-treatment with chemotherapy and gefitinib (Song et al., 2019). Multiple randomized controlled phase III studies, such as ENSURE (Wu et al., 2015), OPTIMAL (Zhou et al., 2011), and EURTAC (Rosell et al., 2012), have shown that erlotinib exhibited better PFS, ORR, quality of life, and tolerable than traditional chemotherapy for patients with EGFR-sensitive mutations. Icotinib, as a first-generation EGFR-TKI developed in China, demonstrated similar results as gefitinib and erlotinib in patients with EGFR-sensitive mutations, which has been approved by the National Medical Products Administration (NMPA) in 2011 for the treatment of patients with advanced EGFR-sensitive mutation NSCLC (Shi et al., 2013). Afatinib, as an irreversible second-generation EGFR-TKI, showed significant improvement in PFS and ORR in EGFR-sensitive mutation NSCLC when compared with gefitinib, but there was no significant difference in OS (Paz-Ares et al., 2017). Dacomitinib, as another second-generation EGFR-TKI, also showed the same benefit in clinical trials and real world practice. Although the second-generation TKIs could provide better efficacy, it lacks selectivity. Not only does it affect the mutant EGFR site but it also affects the wild-type EGFR, which induced the high incidence of adverse reactions. Unfortunately, drug resistance and disease progression are observed after a few months of treatment with the first- or second-generation EGFR-TKIs. About 60% of patients developed p. T790M mutation, which could prevent the first- or second-generation EGFR-TKIs from binding to the receptor (Sequist et al., 2011). In order to overcome this problem, irreversible EGFR-TKIs have been developed to enhance the inhibitory effect on tumors and eliminate the acquired resistance brought by mutations through covalent binding with sites. Osimertinib, as a selective ATP inhibitor, was the most important member of third-generation TKIs. It can selectively respond to the p. T790M mutation, thus avoiding the negative effects on wild-type EGFRs and demonstrating excellent ORR and PFS in p. T790M patients (Piotrowska et al., 2018). Importantly, osimertinib has also been approved as a first-line treatment for EGFR-sensitive mutation. In addition, almonertinib and furmonertinib have also been approved for EGFR p. T790M patients by the NMPA. Unfortunately, it has been reported that the third-generation EGFR-TKIs will also be accompanied by acquired resistance, and C797S mutation is the major resistance mechanism (Thress et al., 2015; Oberndorfer and Müllauer, 2018). Encouraging results have demonstrated that the fourth-generation EGFR-TKIs achieve a desirable efficacy in C797S mutation mouse models when combined with cetuximab (Wang et al., 2017). In the future, more and more selective TKIs will be developed to extend the survival time, and the NSCLC patients with EGFR-sensitive mutation would achieve a long journey of targeted therapy with TKIs generation-by-generation.

### Treatment With ALK-TKIs

Rearrangement of ALK accounts for 4%–7% of NSCLC patients, only second to EGFR mutation, which promotes the growth and proliferation of malignant tumor cells (Soda et al., 2007). Based on the positive results of the study PROFILE 1014, crizotinib was recommended as the first-line treatment for ALK-positive NSCLC (Solomon et al., 2016). Statistics showed that the median PFS (10.9 vs. 7.0 months) and ORR (74% vs. 45%) were all significantly improved in crizotinib-treated patients when compared with conventional platinum doublet chemotherapy (Solomon et al., 2018b; Peters and Zimmermann, 2018). Although visual disturbances (photopsia and blurred vision) and gastrointestinal side effects were observed in some patients after exposure to crizotinib, most of which were slight and tolerable. As a multi-target TKI, crizotinib was not only widely used in ALK-positive mutation NSCLC patients but also showed a good therapeutic effect in other mutations such as ROS-1 and MET.

With the development of tumor-related small-molecule targeted therapy, a second-generation ALK-TKI alectinib significantly increased the median PFS (25.7 vs. 10.4 months) than crizotinib in the first-line treatment of ALK-positive NSCLC (Mok et al., 2020). Meanwhile, alectinib, ceritinib, and brigatinib all could overcome the resistance resulting from crizotinib treatment. Similar to EGFR-TKIs, the treatment of ALK-TKIs also showed inevitable drug resistance after a long time, which drove the development of new TKIs. Lorlatinib, as the unique third-generation ALK-TKIs, when selected as the first-line treatment, achieved 90% ORR from all NSCLC patients and 66.7% ORR from the patients with central nervous system metastases (Solomon et al., 2018a). Up to date, several kinds of resistance mechanisms of ALK-TKIs have been discovered, including ALK amplification, EGFR/HER1, HER2 and HER3 upregulation, cKIT amplification, and various ALK mutations including L1196M (Doebele et al., 2012; Katayama et al., 2012; Tanizaki et al., 2012; Kim et al., 2013). New generations of the ALK inhibitor, TPX-0131, could bind completely within the ATP binding boundary to overcome various ALK-resistant mutations, especially compound mutations L1196M/G1202R and SFM G1202R (Cui et al., 2020). At present, a phase I/II trial (NCT04849273) is being conducted to evaluate patients with ALK-positive advanced or metastatic NSCLC.

## Immunotherapy in NSCLC

Immunotherapy depends on the activation of T cells from the inhibitory status and functional exhaustion to eliminate tumor cells, which could be promoted by some tumor-infiltrating lymphocytes. However, some lymphocytes attack cancer cells or promote the immune escape of cancer cells (Bremnes et al., 2011; Tu et al., 2020). The abnormal expression of immune checkpoint is one of the mechanisms of tumor immune escape. Therefore, immune checkpoint inhibition approaches have been approved for the treatment of cancer in recent decades.

### Treatment With PD-1 Inhibitors

As the first PD-1 inhibitor worldwide, nivolumab was reported with better clinical results than docetaxel in advanced NSCLC (median OS 9.2 vs. 6.0 months, response rate 20% vs. 9%, and median PFS 3.5 vs. 2.8 months in pretreated squamous NSCLC) (Pardoll, 2012). In addition, it also showed that 7% of the patients had grade 3 and 4 toxicities when administrated at a dose of 3 mg/kg of body weight every 2 weeks, much less than 55% of patients treated with docetaxel at 75 mg/m^2^ of the body surface area every 3 weeks. The positive result of CheckMate 017 prompted the FDA to approve nivolumab for the treatment of NSCLC. In addition, CheckMate 057 demonstrated that nivolumab with longer median OS (12.2 vs. 9.4 months) and 18-month overall survival rate (39%) is better than that of docetaxel (23%). Nivolumab did not show a good PFS (2.3 months vs. 4.2 months), but it was superior in 1-year PFS (19 vs. 8%) (Park et al., 2018). CheckMate 057 concluded that immunotherapy with nivolumab achieved good clinical efficacy in NSCLC, and the PD-L1 biomarker expression was positively correlated with the efficacy.

Pembrolizumab showed better efficacy than docetaxel at different doses in phase III KEYNOTE-010. Although there was no significant difference between the two drugs in the median PFS, the median OS of pembrolizumab (10.4 months at low doses and 12.7 months at high doses) was longer than that of docetaxel (8.5 months) (Pauken and Wherry, 2015). Pembrolizumab showed a superior response in patients with the PD-L1 expression of at least 50% from tumor cells, and the median OS was 14.9 months at low doses and 17.3 months at high doses, compared with 8.2 months of docetaxel. At the same time, the median PFS was significantly increased, which reached 5.0 and 5.2 months at low doses and high doses, respectively. The median PFS of docetaxel was only 4.1 months. Also, pembrolizumab monotherapy improved the OS as first-line monotherapy in locally advanced or metastatic NSCLC patients expressing PD-L1 at least 1% from tumor cells (Paz-Ares et al., 2019). Except for monotherapy, the combination therapy was also approved for pembrolizumab. Based on the positive results of KEYNOTE-189 and KEYNOTE-407, the combination treatment of chemotherapy and pembrolizumab was approved for the first treatment of advanced adenocarcinoma and squamous cell carcinoma of NSCLC, especially for those with negative or low expressions of PD-L1. Regardless of monotherapy or combination therapy, pembrolizumab-induced immune-related adverse reactions were all controllable.

In China, there are some PD-1 inhibitors that have been approved by the National Medical Products Administration (NMPA), such as sintilimab, camrelizumab, sugemalimab, and tislelizumab. Those PD-1 inhibitors have demonstrated inspiring efficacy in advanced NSCLC as monotherapy or in combination with standard platinum doublet chemotherapy (Yy et al., 2020; Zhou et al., 2020; Zhou et al., 2021a; Zhou et al., 2021b; Lu et al., 2021; Wang et al., 2021). Serplulimab was approved for those MSI-H, unresectable, metastatic malignant or failed from standard therapy solid tumors, including NSCLC. So far, PD-1 inhibitors have shown remarkable clinical efficacy, thus being considered a revolutionary breakthrough in the treatment of NSCLC at present.

### Treatment With PD-L1 Inhibitors

PD-L1 inhibitor is another important immune checkpoint inhibitor, which has been approved for various solid tumors, front-line or multi-line therapy. The POPLAR study has reported that atezolizumab exhibited better efficacy than traditional chemotherapy (docetaxel) in advanced NSCLC. The OS was 12.6 months for the atezolizumab-treated group, which is longer than 9.7 months for the docetaxel group. Importantly, the results also showed that the OS was positively associated with the expression of PD-L1. The adverse events were observed in 8% of patients with atezolizumab and 22% of patients with docetaxel (Fehrenbacher et al., 2016). Except for atezolizumab, durvalumab is another important PD-L1 inhibitor, which has been approved for the first-line treatment of SCLC based on the positive results of the CASPIAN study. Furthermore, the clinical study of durvalumab in the treatment of advanced NSCLC is still being explored. Envafolimab, the world’s first subcutaneous PD-L1 inhibitor, has been approved for the second-line treatment of advanced MSI-H/dMMR colorectal cancer, stomach cancer, and other solid tumors (including NSCLC) by NMPA in 2021.

## Combination of TKIs and PD-1/PD-L1 in Lung Cancer

Previous studies have reported that an immune inhibitory effect was observed in the EGFR mutation status. Not only Tregs and MDSCs but also TILs, TAMs, immune cytokine, and exosomes were studied as sheets for the dynamic changes in the TME (Huang et al., 2013; Zhang et al., 2014; Gainor et al., 2016; Mascia et al., 2016; Dong et al., 2017; Mazzaschi et al., 2018; Park et al., 2018; Jia et al., 2019a; Poggio et al., 2019). Unique TME from NSCLC patients who harbor EGFR mutations is different from EGFR wild-type patients, and the gene mutation status could influence the immune system with the change in the TME (Lin A. et al., 2019).

Early pre-clinical studies have shown that EGFR-sensitive mutation could increase the expressions of PD-1, PD-L1, and CTLA-4 through the P-ErK1/2P-C-Jun signaling pathway and accelerate the apoptosis of T cells in the TME (Akbay et al., 2013; Chen et al., 2015; Ota et al., 2015), which induce the immune escape of tumor cells, suppressing the body’s own immune function. This phenomenon was observed in mice and EGFR-mutated mouse models when treated with anti-PD-L1 antibodies. The immunohistochemical analysis of PD-L1 expression in the samples of patients with surgically resected NSCLC also showed that EGFR mutation and adenocarcinoma were independent factors of increased PD-L1 expression (Azuma et al., 2014). In addition, other studies have also shown that the expression of PD-L1 was significantly increased in patients with ALK-positive mutation.

### Treatment With TKIs Combined With ICIs

In a phase I/II clinical trial, crizotinib combined with nivolumab was used as a first-line treatment for advanced NSCLC patients with ALK-positive mutation, while multiple cases of severe hepatotoxic adverse reactions were reported. According to a safety review reported in November 2016, liver toxicity greater than grade 3 occurred among 3 of 13 patients, which resulted in the discontinuation of study (Spigel et al., 2018). After that, additional two patients developed ≥ grade 3 liver toxicity, and both of these patients died in the end. These frustrating results indicated that the severe liver toxicity could induce worse outcomes, and the rest of the patients discontinued the combination therapy.

The CAURAL study was a randomized phase III trial to compare the efficacy of osimertinib combined with durvalumab and osimertinib monotherapy in advanced NSCLC patients with p. T790M-positive mutation after first-line treatment (Yang et al., 2019). Unfortunately, this study was prematurely terminated due to the safety concerns from another study TATTON (Ahn et al., 2016) as a high incidence of interstitial lung disease (ILD) was found in the arm of osimertinib combined with durvalumab (TATTON). In the CAURAL study, the most common adverse reaction in the combination group (osimertinib combined with durvalumab) was rash (67%), yet no adverse reactions greater than grade 3 or 4 was reported. Meanwhile, one patient who had only one dose of durvalumab developed grade 2 ILD within osimertinib monotherapy, which may be related to the treatment of osimertinib. The incidence of ILD was 38% (13 cases out of 34) in TATTON, whose data were significantly higher than the incidence of ILD with osimertinib (2.9%) or durvalumab (2%) monotherapy. Comparing the data obtained from these two trials, there was no significant difference in drug dosage or race for the study population. Although the incidence of ILD with the EGFR wild-type in the TATTON experiment is relatively high (64%), those results cannot be used as confirmative evidence due to the small sample size of the study. ILD has not been found in the treatment of erlotinib and gefitinib which were combined with durvalumab or atezolizumab. At present, only the combination of osimertinib and durvalumab could be considered as a cause of the high incidence of ILD. It is worth mentioning that other TKIs could cause adverse events such as hepatotoxicity or fever when combined with ICIs.

Moreover, the combination therapy of gefitinib and durvalumab in advanced NSCLC patients with EGFR-sensitive mutations (19 deletions and 21 L858R) showed grade 3 or 4 elevated levels of AST (70% in arm 1, 60% in arm 2) and ALT (40% in arm 1, 50% in arm 2), but both events could be controlled after the drug withdrawal or administration of corticosteroids (Gibbons et al., 2016). Given that ORR of arm 1 is 77.8% and 80% of arm 2, the combination therapy was worthy of being further studied at the full dose. In addition, there are clinical trials that have investigated the combination of erlotinib and ICIs in NSCLC patients. In the phase I study of NCT02013219, erlotinib combined with atezolizumab in EGFR mutation-positive (19 deletions and 21 L858R) advanced NSCLC patients who did not use TKI achieved 75% ORR (Ma et al., 2016a). At the same time, 39% of the patients had grade 3 or higher adverse reactions, among which the most common reactions were fever and ALT elevation, and there were some cases of drug withdrawal due to those adverse reactions. The combination therapy increased the incidence of toxic and side effects, but the efficacy was not significantly improved. In a phase II study, 21 advanced NSCLC patients with EGFR-mutated (19 deletions and 21 L858R) were enrolled, and the patients received the combination treatment of nivolumab and erlotinib with 15% ORR and 65% DCR (Hellmann et al., 2017). Unfortunately, there was no significant improvement in efficacy, but the toxicity was significantly increased. At present, the combination therapies of ICIs and TKIs are not approved in clinical practice yet. The mechanisms of treatment effects and adverse reactions need to be further and clearly investigated. An overview of the studies is shown in Table 1.

**TABLE 1:** Prospective studies evaluating the efficacy of combination or sequential ICIs and TKIs.

Study	No. and patients	Combination	Targeted agent	Immunotherapy	Phase	Status
CheckMate-370 Spigel et al. (2018)	13, newly diagnosed/maintenance LA/stage IV NSCLC	Concurrent	Crizotinib	Nivolumab	I/II	Completed
CAURAL Yang et al. (2019)	14, locally advanced or metastatic NSCLC EGFR + p. T790M	Concurrent	Osimertinib	Durvalumab	III	Active, not recruiting
TATTON Ahn et al. (2016)	11, advanced non–small cell lung cancer	Concurrent	Osimertinib	Durvalumab	I	Active, not recruiting
NCT02088112 Gibbons et al. (2016)	Arm 1 10, arm 2 10, carcinoma, non–small cell lung cancer	Concurrent	Gefitinib	Durvalumab	I	Completed
NCT02013219 Ma et al. (2016a)	20, non–small cell lung cancer	Concurrent	Erlotinib	Atezolizumab	I	Completed
CheckMate-012 Hellmann et al. (2017)	21, non–small cell lung cancer	Concurrent	Erlotinib	Nivolumab	I	Completed
JAVELIN 101 Alice and Shaw (2018)	40, non–small cell lung cancer	Concurrent	Crizotinib and lorlatinib	Avelumab	Ib	Completed
Oshima et al. (2018)	20,516, NSCLC	Concurrent and Sequential	EGFR-TKIs	Nivolumab	-	-
Kato et al. (2017)	155, stage IV cancer with post-immunotherapy	Sequential	EGFR-TKIs	CTLA-4, PD-1/PD-L1 inhibitors, or other agents	-	-
Schoenfeld et al. (2019)	126, NSCLC, treated with PD-1/PD-L1 inhibitors and EGFR-TKIs	Concurrent and sequential	EGFR-TKIs	PD-1/PD-L1 inhibitors	-	-
Kotake et al. (2017)	19, NSCLC EGFR + p.T790M	Sequential	Osimertinib	Nivolumab	-	-
Mamesaya et al. (2017)	1, NSCLC EGFR + p.T790M	Sequential	Osimertinib	Nivolumab	-	-
Uchida et al. (2019)	26, treatment with EGFR-TKIs immediately before and/or after PD-1 inhibitors	Sequential	EGFR-TKIs	PD-1 inhibitors	-	-
Lin et al. (2019b)	453, NSCLC, treated with crizotinib	Sequential	Crizotinib	ICIs	-	-

### PD-1/PD-L1 Inhibitor Treatment With TKI Pre-/Post-Treatment

Pre-clinical studies have shown that the intrinsic PD-L1 expression was upregulated in EGFR-sensitive mutation NSCLC cells, which induces the apoptosis of T cells and facilitates the immune escape. In addition, MHCI and class II molecules were enhanced after exposure to EGFR-TKIs, which will always be accompanied by the induction of IFN-γ and T-cell–mediated tumor killing. For progressive patients who have been pretreated with TKIs, sequential treatment with ICIs was widely reported (Yang et al., 2019). KEYNOTE-001, a phase I trial, indicated that pembrolizumab was not suitable for EGFR-positive mutation patients who have been pretreated with TKIs (median OS 4 months and median PFS 5.3 months) (Garon et al., 2019). The curative effect was significantly lower than that of other patients who received TKI treatment naive (median OS 18.6 months and median PFS 4 months). A randomized phase III trial, IMpower 150, investigated the efficacy of atezolizumab combined with bevacizumab and chemotherapy in NSCLC, and key subgroup analyses were conducted for patients with EGFR-sensitive mutations (Reck et al., 2019). It is noteworthy that it showed a favorable response for this combination pattern when compared with bevacizumab combined with the chemotherapy group (median duration of response: 11.1 vs. 5.6 months).

On the other hand, several clinical studies have demonstrated that EGFR/ALK-positive mutation NSCLC was not suitable for ICI monotherapy or TKIs combined with ICIs. In advanced NSCLC patients with sensitive mutations, the response rate of PD-1 inhibitors was less than 5%, while the response rate of corresponding TKIs was 70%. In the combination therapy of ICIs and EGFR-TKIs, the incidence of side effects was significantly increased (Hsu et al., 2019). A retrospective study conducted in Japan showed that among 20,516 advanced NSCLC patients with EGFR-sensitive mutation, the total incidence rate of ILD or immune pneumonia was 4.8%; the incidence rates of pneumonia were 4.6% and 6.4% when treated with TKIs or nivolumab, respectively, and 25.7% in the combination therapy. When further stratified the patients by treatment with and without nivolumab, the odds ratios of EGFR-TKI-associated immune pneumonia in cases with and without nivolumab treatment were 5.09 and 1.22, respectively (Oshima et al., 2018). Nivolumab even developed explosive disease progression in two patients with lung adenocarcinoma who were resistant to chemotherapy and EGFR-TKI therapy (out of 155 participants). Within 2 months, the tumors increased by 53.6% and 125%, respectively, several times faster than before ICI was introduced (Kato et al., 2017). In other words, high risk and probability of adverse events severely limit the options for the sequential therapy of ICIs and TKIs.

In clinical practice, it is often the case that a short time of immunotherapy or immunochemotherapy was conducted at first, and then, patients were changed to small-molecule targeted therapy. This is due to the fact that the advanced NSCLC patients often need an urgent therapy to control the progression of disease and fill the gap while waiting for the genetic test results. Schoenfeld et al. (2019) have reported that in NSCLC patients with EGFR-sensitive mutations, the treatment of osimertinib within 3 months after ICIs increased the incidence of grades 3–5 adverse reactions (interstitial pneumonia or enterocolitis). By contrast, no severe adverse reactions were observed among patients treated with either osimertinib followed by PD-1/PD-L1 (0 of 29) or PD-1/PD-L1 followed by other EGFR-TKIs (afatinib or erlotinib, 0 of 27). It was considered that it appears to be drug-specific, rather than class-specific, the interaction between osimertinib and PD-1 inhibitors. In addition, there were also a number of other studies on the sequential treatment with osimertinib and ICIs. Both Kotake et al. (2017) and Mamesaya et al. (2017) have all found a high incidence of interstitial pneumonia in retrospective case collections with nivolumab prior to osimertinib. Uchida et al. (2019) have reported the same phenomenon that interstitial pneumonia occurred in the sequential treatment of ICIs followed by osimertinib; however, it was not the case in the sequential treatment of ICIs followed by first- or second-generation EGFR-TKIs. The administration of osimertinib immediately after treatment with PD-1 inhibitors was observed in three patients. In addition, Oshima et al. (2018) have investigated the relationship between the types of ICIs and the occurrence of interstitial pneumonia. In comparison with monotherapy, a higher proportion of interstitial pneumonia was observed for concurrent or sequential treatment of nivolumab and EGFR-TKIs. Lin JJ. et al. (2019) have also reported that the risk of hepatotoxicity morbidity experienced an increase in their analysis in a series of patients who received immunotherapy before crizotinib. Jia et al. (2019b) have demonstrated that the combination treatment of EGFR-TKIs and ICIs may induce overlapping toxicities in an EGFR-mutated mouse model. Based on these findings, we should soberly realize that when TKIs were combined with ICIs, the sequence and the timing may influence the severity of pneumonitis. An overview of the studies mentioned earlier is shown in Table 1.

## Discussion

Nowadays, accumulating therapies with efficacy are available clinically for patients with NSCLC. Apart from surgery and conventional chemotherapy, targeted therapies could achieve significant efficacy and low adverse events and improve the quality of life for patients with specific genetic mutations. The particular system of targeted therapies elucidates the possibility of long-term treatment in the future, yet the development of new generations of targeted TKIs to overcome the acquired resistance is still an urgent task. Immunotherapy, as a novel therapy for the treatment of NSCLC in recent years, has demonstrated significant efficacy in clinical practice as monotherapy or combined with chemotherapy, and higher ORR will be achieved in long-term treatment. Several clinical trials are exploring the clinical application of ICIs at different stages of NSCLC as monotherapy or combination therapy. As of 2021, there are thousands of clinical trials at *clinicaltrials.gov* about different kinds of ICIs for the treatment of lung cancer, such as pembrolizumab, nivolumab, and atezolizumab . Although a long-term survival could be achieved for ICIs, a series of immune-related adverse events could not be ignored. It has been reported that the activation of the oncogenic EGFR pathway enhances the susceptibility of the lung tumors to PD-1 blockade in the mouse model, suggesting the combination of the PD1 blockade with EGFR TKIs may be a promising therapeutic strategy. Hence, in order to acquire maximum benefit for patients, the combination of TKIs and ICIs has been explored in previous clinical studies. Unfortunately, when small-molecule TKIs were combined with ICIs, the original treatment effect was not significantly improved, whereas the probability of grade 3 and 4 adverse reactions was increased. In the TATTON study, the combination use of osimertinib and durvalumab induces the high incidence of interstitial lung disease, which led to the mandatory discontinuation of several similar clinical studies (Ahn et al., 2016). The combination of gefitinib combined with durvalumab demonstrated encouraging activity but higher incidence of grade 3/4 liver enzyme elevation (40–70%) (Gibbons et al., 2016). The treatment-related grade 3–4 adverse events were observed in 39% of patients when treated with atezolizumab combined with erlotinib (Ma et al., 2016b). The phase 1b JAVELIN 101 Lung trial evaluated the second-line combination of avelumab and crizotinib in ALK-negative NSCLC patients, and 2 out of 12 patients (16.7%) had dose-limiting hepatotoxicity. Other notable dose-limiting toxicities included rash, febrile neutropenia, and QT prolongation. However, in the cohort of ALK-translocation–positive NSCLC in this study, no dose-limiting toxicities were observed when avelumab was combined with lorlatinib (Alice and Shaw, 2018). Numerous studies have shown that the expression of TME and PD-L1 will be affected by pretreatment with TKIs, thus affecting the efficacy of immunotherapy. Previous studies have reported that the high expression of PD-L1 may be related to the acquired resistance of EGFR-TKI. Hence, EGFR-TKI may not be suitable for patients with wild-type EGFR or high expressions of PD-L1 (Su et al., 2018; Hsu et al., 2019). The changes in the TME may influence the selection of EGFR-TKIs and ICI combination therapy, and the development of the TME during treatment may also render the most effective treatment (Gettinger et al., 2018; Yoshida et al., 2018; Yamada et al., 2019). It is possible to dynamically monitor the immune activity of the TME for a long time to maximize the impact of immunotherapy on patients. Based on previous studies, the safety profiles associated with concurrent TKIs and ICIs are quite variable among studies. It is of note that most of these combinations have generally shown somewhat higher toxicity than expected, and this unexpected high incidence of adverse events results in the limitation to further active investigation. Also, it reflects the potential exacerbation of intrinsic but typically minimal toxicities of various TKIs. To the best of our knowledge, no concurrent combination therapy of TKIs and ICI phase 3 clinical trial in TKI-naive patients is currently planned or actively accruing.

In addition to concurrent therapy, sequential therapy is also another important pattern of combination therapy. As it is known to us, the prior selection for patients with sensitive mutation (EGFR/ALK) is still TKIs. However, acquired resistance is inevitable and severely limits its clinical application. Based on the positive results of IMpower 150 and other phaseⅠ/Ⅱ trials, this sensitive mutation (EGFR/ALK) NSCLC will always conduct immunotherapy (monotherapy or combined with chemotherapy) after failure from first-/second-line targeted therapy. The sequential treatment as ICI pre-treatment with TKIs is the most common pattern in clinical practice, and ICI post-treatment with TKIs also exists. Most of the sequential treatment of TKIs and ICIs could achieve a longer OS, and drug-induced toxicity is tolerable. However, a previous study has also shown that ILD was observed in the treatment sequence of an anti-PD-1 antibody followed by osimertinib but not with first- or second-generation EGFR-TKIs. Jia et al. (2019b) have reported that osimertinib, rather than gefitinib combined with anti-PD-L1 treatment, could lead to lung injury in an EGFR-mutated tumor-bearing mouse model. This may be due to the durable immune response of the PD-(L)1 antibody. In contrast, EGFR-TKIs such as osimertinib take effect in a short period of time. They also speculated that the mechanism of ILD development is different between first-/second-generation TKIs and osimertinib, and the activation of T-cell effects by ICIs may upregulate this effect synergistically to cause ILD with osimertinib but not first- or second-generation TKIs. Jia et al. (2019a) have also reported that EGFR-targeted therapy alters the tumor microenvironment in EGFR-driven lung tumors. The optimal sequence of the treatment and strategies that modulate the tumor microenvironment to a state that may favor antitumor immune responses need to be considered when designing clinical trials. As it is known to us, the treatment of NSCLC is a “multi-station” manner, including the sequential therapy of TKIs and ICIs, which could provide a pivotal benefit for advanced NSCLC. However, further studies are still needed to explore the mechanism of adverse events and optimal sequence of the treatment and strategies.

In conclusion, the combination treatment of EGFR/ALK-TKIs and ICIs in NSCLC should be considered investigational; the specific drug, optimal dosing, sequence of the treatment schedule, interval time, treatment-related toxicities, and efficiency should all be considered in this type of combination therapy.
